# Bank behavior due to a buildup in Chinese nonperforming loans

**DOI:** 10.1371/journal.pone.0295667

**Published:** 2023-12-07

**Authors:** Yuchi Xie, Qizhen Ye, Raofeng Guo

**Affiliations:** 1 Faculty of Professional Finance and Accountancy, Shanghai Business School, Shanghai, China; 2 Shanghai University of Finance and Economics, Shanghai, China; Centre for Studies in Social Sciences, Calcutta, INDIA

## Abstract

We study 33 Chinese banks from December 2009 to December 2020 to examine the moral hazard behavior of Chinese banks from the perspective of nonperforming loans (NPLs). The results show that banks with low returns are more likely to engage in risk-taking behavior and have a high number of NPLs. The government could adopt regulations that would help joint stock banks to be more prudential and encourage city and rural banks to take more risks.

## Introduction

With the rising risk related to real estate, the non-performing loans of Chinese commercial banks have received great attention. The rise of non-performing loans not only is becoming a potential risk for the Chinese financial system but also adversely affecting the bank’s lending operations. In addition, the feedback effects of NPLs from the banking system to economic activity undermine the economy’s ability to sustain growth and increase economic vulnerabilities going forward. Acknowledging the importance of NPLs, policymakers have prioritized the prevention of systemic risk and control over NPLs. At the Chinese Politburo meeting in December 2017, the importance of employing macro leverage to address systemic risk and strengthen the ability of financial institutions to service the real economy was articulated (The direction of Chinese economic policy is typically set at meetings of China’s supreme ruling body, the Politburo). Several goals were outlined, such as detecting risky investments, preventing asset bubbles, and improving oversight. The increase in NPLs poses a big challenge in terms of controlling Chinese financial risk. Adequately dealing with NPLs helps to prevent an outbreak of systemic financial risk and maintain steady growth in the Chinese economy.

NPLs result from poor investment and business operations, which are closely related to the Chinese economic development stages and models. After several decades of rapid economic growth, China has entered a new stage of slower growth. A growth pattern driven by exports and investment is no longer sustainable. The industries that previously supported China’s economic growth have gradually lost their competitiveness, as they are usually less productive. They can also crowd out investment in new industries, which not only increases the probability that banks will have a high number of NPLs but also prevents the development of new industries and the economy. The slowdown in economic growth puts downward pressure on aggregate demand and production, which results in lower expected returns, loss of profits, and an accumulation of NPLs at banks.

As part of banking reforms, the Chinese government introduced competition into the banking system by privatizing state-owned banks. In 2004, China began a program of "equitization" and transformed state-owned banks into joint-stock banks. The goal of this transition is to increase the incentives for banks to operate as profit-driven firms with less control by the government. Despite this change, the largest shareholders of Chinese joint stock banks remain state-owned enterprises (SOEs) [1].

Based on the availability of data, this paper studies thirty-three banks listed on the Shanghai and Hong Kong stock exchanges from 2009 to 2020. The variables used to demonstrate bank performance are lagged NPLs, the loan growth rate, the provision coverage ratio, the debt-to-asset ratio, the return on assets, the return on equity, and the economic growth rate. We employ the threshold model used by Zhang et al. to test the moral hazard behavior and threshold effect of the banks [2].

Our major findings show that banks with high debt and low returns are more likely to have moral hazard problems and regulations regarding bank capital help to mitigate these problems. The moral hazard behavior by banks and the slowdown in economic growth increase the number of NPLs. This paper contributes to previous literature by examining the moral hazard behavior at banks to study the determinants of Chinese bank behavior and how government regulations could reduce moral hazard problems.

Our paper is organized as follows: The Literature Review section summarizes the literature. The Data and Models section describes the data and models. The Empirical Regression section presents the empirical results and the Robustness Analysis section provides a robustness analysis. The Conclusion and Policy Implications section summarizes the paper and proposes policy recommendations.

## Literature review

Previous studies have examined the relationship between macroeconomic factors and NPLs by testing whether they are the result of cyclical economic development [3–9]. Ghosh finds a negative relationship between US growth in the gross domestic product (GDP) and NPLs [10]. Balgova, Nies, and Plekhanov find that countries that actively reduce the number of NPLs typically experience higher growth rates [11]. The high economic growth gives firms more room for repaying debt and upgrading production, which not only helps the economy to grow sustainably but also helps to tamp down NPLs.

Berger and DeYoung examine the relationship between NPLs and cost efficiency in US commercial banks from 1985 to 1994, finding bidirectional causality between them [12]. Many papers have studied the moral hazard behavior by banks and its effect on NPLs. Several papers focus on the relationship between the lending rate and NPLs. Louzis, Vouldis, and Metaxas examine this relationship in Greece and find that a high lending rate could lead to a high number of NPLs [13]. Bofondi and Ropele report similar results in Italy [14].

Keeley argues that Chinese commercial banks, which are insured by the Chinese government, are more likely to take excessive risks [15]. Kornai was the first to propose a soft budget constraint and found that firms with soft budget constraints are more likely to suffer from moral hazard problems. The soft budget constraint is widely cited as the major cause of the high number of Chinese NPLs [16]. Maskin et al. study how a soft budget affects the development of economies in a transition stage and conclude that a soft budget could lead to inefficiency [17]. Podpiera et al. study bad management and its negative effect on the behavior of banks in emerging markets [18]. Shi argues that a soft budget constraint at SOEs and state-owned banks contributes to the high number of Chinese NPLs [19]. As China is in a transition from having a planned economy to a market-based economy, its government allows soft budget constraints at SOEs, which have an incentive to lend more to SOEs driven by their assumed government guarantee.

Prior research shows that a lack of capital could encourage moral hazard behavior [20, 21]. Bernanke and Gertler argue that prudential banks are careful to avoid having an excessively high NPL ratio [22]. However, after the NPL ratio reaches a high level, banks become more aggressive and tend to take more risk. Keeton and Morris state that banks with relatively low capital respond to moral hazard incentives by increasing the riskiness of their loan portfolio, resulting in a higher number of NPLs [23]. Other papers examine the role played by capital regulation in mitigating moral hazard problems that are due to a lack of capital [24–27]. Kim et al. examine the effect of deposit insurance on banks and conclude that banks tend to engage in moral hazard behavior with deposit insurance [28]. Klomp et al. find that stricter regulation could reduce banking risk given high institutional quality [27]. The model by Allen et al. shows that capital regulation with insured deposits not only can help the government reduce bankruptcy costs but also achieve higher social welfare [29]. Tanaka et al. study the optimal ex-ante capital and find that banks with low returns and high debt are inclined to have ex-ante moral hazard problems, which leaves room for the government to intervene to prevent further losses [30].

Zhang et al. find that the rise in the NPL ratio in China increases risky lending [2]. They use a threshold model to find the threshold value of NPLs and the capital adequacy ratio (CAR), which helps the government to set the capital requirement for preventing moral hazard behavior due to risky lending. Li found that banks with great market power are less likely to engage in risk-seeking activity [31]. We test the moral hazard behavior of Chinese banks by focusing on how bank performance affects NPLs and the role played by the government in mitigating the moral hazard problems. When banks have a high level of impaired loans, they have a greater incentive to engage in risky investments to compensate the losses of loans [32, 33]. We include lagged NPLs in our model to test the effect of the prior period of NPLs on bank decision-making. Besides, we assume that banks with high debt and low returns are more likely to engage in risky investment, which yields high returns in the short term but is harmful in the long term. The decisions of lending are based on the potential returns of a loan and the profit of a loan is largely determined by the revenue of the project. Credit managers of banks could estimate the risk of default based on the forecast of revenues and valuation of collaterals. The low return from projects puts downward pressure on the revenue of banks, which could result in the risk-seeking behavior of bank managers to offset the losses. We also assume that the Chinese government could mitigate the moral hazard behavior by imposing regulations.

Mohapatra et al. study how economic growth affects the system risk and the relationship between the competition of banks and systemic risk. They find that healthy competition helps the development of banks, while fierce competition negatively affects the development of banks [34]. Dong et al. use four types of Chinese commercial banks to study the cost and profit efficiency of Chinese banks and the factors to affect the efficiency of Chinese banks. They find that the shadow return on equity could result in less efficiency of Chinese banks [35]. Rahman, M. R. uses the Indian industry as a case to study the effectiveness of management [36].

This paper differs from a previous paper in the following aspects: First, this paper employs a threshold regression model based on Hansen to test whether banks with some NPLs below the threshold level tend to take more risk than those with the number of NPLs above the threshold level and tend to have more moral hazard problems. Second, this paper tests whether banks with high debt and low return tend to take more risk than banks with low debt and high return. Third, this paper tests whether government regulations help to alleviate moral hazard behavior.

## Data and models

Given the data availability, this paper studies 33 banks from 2009 to 2020, with a total number of 396 observations (The 33 banks consist of 14 joint stock banks and 19 city and rural banks. The capital of JSBs is partly helped by the state and some are invested by foreign investors. A large proportion of CRBs’ security held is domestic entities and the major function of CRBs is to provide financial services to local businesses.). The NPLs, the loan growth rate (LGR), the provision coverage ratio (PCR), the debt-to-asset ratio (DAR), the return on assets (ROA), and the return on equity (ROE) for the 33 banks come from CSMAR (China Stock Market & Accounting Research) database(The CSMAR database offers financial statements by China’s listed companies)and the Wind terminal(Wind terminal is China’s financial database and software). China’s growth rate of the gross domestic product (GDP) comes from the National Bureau of Statistics of China.

It is well demonstrated in the literature that a high LGR can lead to excessively risky investment and a high number of NPLs. Berger and DeYoung focused on cost efficiency in US commercial banks from 1985 to 1994 to test the moral hazard behavior of banks, finding bidirectional causality between cost efficiency and NPLs [12]. They propose poor managerial efficiency and explain why banks choose investment projects with little credit, which finally turn out to be non-performing loans. Our arguments follow the moral hazard hypothesis by Zhang et al. that banks with high previous losses tend to increase loans and take excessive risk, which could lead to a high level of NPLs [2].

This paper uses the PCR and the CAR to proxy for government regulations on capital. PCR refers to the ratio of loan loss reserves against the balance of non-performance loans. This ratio is set up by the China Banking Regulatory Commission (CBRC) to evaluate whether loan loss reserves of commercial banks are enough. The basic standard for PCR is 150%. The lower PCR will leave a large amount of NPLs uncovered, which adds risk to the bank system. Before Feb.28th 2018, the threshold ratio that the CBRC set for banks are 150%, and lower than this threshold is subjected to the punishment from central bank. After Feb. 28th 2018, CBRC reduced the threshold ratio to 120%. The regulation effect of government risk control policy works through two channels. The first channel is the function of PCR, which is used to cover the loan loss. The increase in PCR reduces the probability of capital shortage if there were firm defaults. The second channel is through the punishment effect from CBRC. The punishment not only results in the capital loss of the bank but also can erode the credibility of a bank, which could result in the loss of highly qualified debtors and creditors. To avoid punishment from CBRC, the banks not only have to avoid risk-seeking behavior but also keep enough PCR to cover the loan loss. Therefore, the threshold of PCR setting by CBRC could regulate bank operation behavior through the function of PCR and punishment from CBRC. The CAR is the ratio of a bank’s available capital to its risk-weighted credit exposure. The China Banking Regulatory Commission (CBRC) sets the threshold of CAR to be 8%. A bank with a high CAR has a large enough cushion to absorb losses and therefore fewer NPLs.

The DAR denotes bank debt. The ROA and ROE measure bank returns. The difference between them is that ROA allows for debt, whereas ROE does not. DAR, ROA, and ROE are used to examine whether banks with high debt and low returns are more likely to suffer large losses [37]. The economic growth rate is employed to study whether a high number of NPLs results from low economic growth.

The statistical descriptions of these variables are in Table 1. The NPLs of most Chinese banks are strictly under control, with an average of 1.24%. The average PCR is 268.64%, which is above the 150% set as the threshold ratio for banks by the CBRC before February 28, 2018. The average GDP growth rate is 7.4%, which shows strong economic growth. The average rate of loan growth is 19.2%, and the average ratio of debt to assets is over 93.2%. The average ROA is 0.9%, and the average ROE is 14.3%.

**Table 1:** Descriptions of data.

Panel A					
**Variables**	**Observations**	**Mean**	**Standard Deviation**	**Min.**	**Max.**
**NPLs**	396	1.239%	0.697%	0.006%	7.7%
**LGR**	396	0.192	0.111	0.033	1.007
**PCR**	396	2.686	1.556	1.049	13.981
**CAR**	396	12.774%	1.642%	8.09%	17.52%
**DAR**	396	0.932	0.013	0.889	0.967
**ROA**	396	0.009	0.003	-0.006	0.027
**ROE**	396	0.143	0.049	-0.083	0.408
**CRR**	396	0.426	0.586	0.148	4.627
**GDP**	396	7.375%	2.012%	2.3%	10.6%
Panel B					
**Variables**		**Definition**		**Data Source**	
**NPLs**	Nonperforming loans	Loans that are subject to late repayment		CSMAR	
**LGR**	Loan growth rate	Percentage change of loan		CSMAR	
**PCR**	Provision coverage ratio	The ratio of loan-loss reserves to the balance of NPLs		CSMAR	
**CAR**	Capital adequacy ratio	The ratio of a bank’s available capital to its risk-weighted credit exposure		CSMAR	
**DAR**	Debt asset ratio	The ratio of total debt to total assets		CSMAR	
**ROA**	Return on assets	The ratio of net income to total assets		CSMAR	
**ROE**	Return on equity	Ratio of net income to shareholders’ equity		CSMAR	
**CRR**	Cost-to-return ratio	The ratio of return to cost		CSMAR	
**GDP**	Gross domestic product	The total value of all the finished goods and services		National Bureau of Statistics of China	

To investigate bank behavior, we employ a threshold regression model based on Hansen [38]. We follow Zhang et al. to use single threshold regression to test the moral hazard behavior of banks.

$$Y_{it}=\alpha_{it}+\beta_{0}{x_{it}I(q}_{it}\leq \gamma )+\beta_{1}{x_{it}I(q}_{it}>\gamma )+z_{it}+\varepsilon_{it},\varepsilon_{it}∼N(0,1)$$ (1)

where *I*(*) is the indicator function, which takes a value of one if the statement in parentheses is true, and, otherwise, zero; *x*_*it*_ are regime-dependent variables, and *Z*_*it*_ is regime-independent variables. *ε*_*it*_ are residuals.

Banks with higher NPLs are expected to take more risks compared with banks with fewer NPLs to reduce the NPLs. Therefore, banks with high NPLs are more likely to participate in risky behavior. Following Zhang et al., we examine moral hazard behavior by testing whether banks with high previous loan losses tend to increase their subprime loans, which leads to large losses in the long run [2]. We include LGR and one-year-lagged LGR in our model to test the moral hazard behavior of banks. The threshold panel regression is used to test the following hypotheses:

H0: Banks with NPLs above the threshold level tend to take more risk and have moral hazard problems.

H1: Banks with NPLs below the threshold level are less likely to have more moral hazard problems.

$${NPL}_{it}=\alpha +\beta_{0}{LGR}_{it}I({l.NPLs}_{it}\leq \gamma ){+\beta }_{1}{LGR}_{it}I({l.NPLs}_{it}>\gamma )+\delta_{it}{DAR}_{it}+\eta_{it}{ROA}_{it}+\rho_{it}{ROE}_{it}+\upsilon_{it}{GDP}_{t}+\varepsilon_{it}$$ (2)

PCR and CAR are employed as threshold variables to test the threshold effects of government regulation on NPLs. Regulators could monitor the PCR and CAR levels to regulate bank behavior and reduce their losses.

Instead of using multiple thresholds regression, the single threshold regression is employed to examine the behavior of banks in two regimes of observation, which are classified based on the assumption of the moral hazard behavior of banks. The following hypothesis is tested using single threshold regression:

H0: Banks with high debt and low return tend to have more NPLs. High PCA and CAR help banks alleviate the pressure of high NPLs.

H1: Banks with low debt and high return tend to have fewer NPLs. Low PCA and CAR add pressure on banks in reducing the level of NPLs.

Eqs (3) and (4) are used to test the effect of regulations on bank behavior.

$${NPL}_{it}=\alpha +\beta_{0}{LGR}_{it}I({PCR}_{it}\leq \gamma ){+\beta }_{1}{LGR}_{it}I({PCR}_{it}>\gamma )+\delta_{it}{DAR}_{it}+\eta_{it}{ROA}_{it}+\rho_{it}{ROE}_{it}+\upsilon_{it}{GDP}_{t}+\varepsilon_{it}$$ (3)

$${NPL}_{it}=\alpha +\beta_{0}{LGR}_{it}I({CAR}_{it}\leq \gamma ){+\beta }_{1}{LGR}_{it}I({CAR}_{it}>\gamma )+\delta_{it}{DAR}_{it}+\eta_{it}{ROA}_{it}+\rho_{it}{ROE}_{it}+\upsilon_{it}{GDP}_{t}+\varepsilon_{it}$$ (4)

where *LGR*_*it*_ is the loan growth rate by bank *i* at time *t*.*l*.*NPLs*_*it*_ is a one-year lag of NPLs by bank *i* at time *t*.*PCR*_*it*_ is the provision coverage ratio by bank *i* at time *t*.*CAR*_*it*_ is the capital adequacy ratio by bank *i* at time *t*.*DAR*_*it*_ is the debt-to-asset ratio by bank *i* at time *t*.*ROA*_*it*_ is the return on assets by bank *i* at time *t*.*ROE*_*it*_ is the return on equity by bank *i* at time *t*.*GDP*_*t*_ is the economic growth rate at time *t*.*ε*_*it*_ are residuals.

## Empirical regression

Because Chinese joint-stock banks (JSBs) operate differently from city and rural banks (CRBs), we divide the banks into these two groups. We include four of China’s four largest commercial banks in the group of JSBs. The JSBs comprise more than half the Chinese banking market and are more likely to be affected by central government economic policy. However, the CRBs are largely affected by local government economic goals and are involved in investment in local projects. Therefore, CRBs tend to lend to enterprises with the involvement of local government. Both JSBs and CRBs play an important but different role in the Chinese banking market. Joint-stock banks typically have above-average credit quality and good geographic diversification in China. They are resilient enough to maintain minimum capital as required by the regulator. Joint-stock banks typically have controllable liquidity risk due to easy access to the interbank market. As the main force of local finance in China, CRBs have played an important role in supporting the development of the local economy. The asset quality and management are less strong than JSBs. The pressure on the regional economy has brought certain constraints to the development of CRBs. The CRBs with good asset quality are mainly concentrated in Zhejiang, Jiangsu, and Guangdong.

Both CRBs and JSBs could have less efficient lending and suffer from moral hazard problems. We first run a series of benchmark linear models and then test the threshold effect for JSBs, CRBs, and all the banks in our sample. *PCR* and *CAR* are both employed as threshold variables to examine moral hazard behavior by banks.

### Fixed-effects regression

We run both fixed- and random-effects models to obtain the Hausman statistic. The p-value of the Hausman statistic is 0.001, which indicates that the use of a fixed-effects model, rather than the random-effects model is more appropriate. The results of the fixed-effects model are in Table 2.

**Table 2:** Fixed-effects regression.

	Model 1	Model 2	Model 3	Model 4	Model 5	Model 6
***LGR***_***it***_	0.002 (0.002)		0.004 (0.002)		0.001 (0.003)	
***L*.*LGR***_***it***_		-0.002 (0.002)		0.002 (0.002)		-0.003 (0.002)
***PCR***_***it***_			-0.002*** (0.0002)	-0.002*** (0.0002)		
***CAR***_***it***_					-0.085*** (0.026)	-0.088*** (0.026)
***DAR***_***it***_	-0.031 (0.050)	-0.029 (0.050)	-0.013 (0.043)	-0.011 (0.043)	-0.089* (0.052)	-0.089* (0.052)
***ROA***_***it***_	-0.872*** (0.324)	-0.927*** (0.324)	-0.780*** (0.280)	-0.795*** (0.280)	-0.804** (0.321)	-0.845*** (0.320)
***ROE***_***it***_	-0.025 (0.019)	-0.024 (0.022)	-0.019 (0.019)	-0.017 (0.019)	-0.028 (0.022)	-0.028 (0.022)
***GDP***_***t***_	-0.040*** (0.013)	-0.036** (0.015)	-0.030** (0.013)	-0.033** (0.013)	-0.047*** (0.014)	-0.043*** (0.015)
***R*** ^**2**^	0.396	0.396	0.552	0.551	0.414	0.416
**Year dummy**	Yes	Yes	Yes	Yes	Yes	Yes
**Bank fixed-effect**	Fixed	Fixed	Fixed	Fixed	Fixed	Fixed
**Observations**	396	396	396	396	396	396

*Notes*: Models 1 and 2 show the regression results of the effect of LGR and L.LGR on Chinese NPLs without the threshold variables. Models 3 and 4 show the results of the effect of LGR and L.LGR on Chinese NPLs with the provision coverage ratio. Models 5 and 6 show the results of the effect of LGR and L.LGR on Chinese NPLs with the capital adequacy ratio. Standard errors are reported in parentheses. ***, **, and * significant at 1%, 5%, and 10%, respectively.

The results in Table 2 show that the response of NPLs to ROA is significantly negative. This result is consistent with our hypothesis that banks with low returns are more likely to have a high number of NPLs. Table 2 also shows that the response of NPLs to PCR and CAR is also significantly negative, which confirms that banks with high loan-loss reserves and more capital reserves are less likely to have high NPLs. The results indicate that the GDP growth rate is negatively related to NPLs, which corroborates that high economic growth helps to reduce NPLs.

### Moral hazard behavior test with NPLs as a threshold variable

To test whether banks with previous losses higher than the threshold value tend to expand loans and have high NPLs, we set *l*.*NPLs*_*it*_ as the threshold value. According to Eq (2), when *l*.*NPLs*_*it*_ exceeds the threshold value, and the effect of *LGR*_*it*_ on *NPLs*_*it*_ is determined by *β*_1_. Banks with *l*.*NPLs* above the threshold value are expected to increase loans and have high NPLs, which means that *β*_1_ should be positive and *β*_0_ should be negative.

To test whether JSBs behave differently from CRBs, we divide the samples into two groups to examine their threshold effects separately. Table 3 reports the results of the threshold effect tests; all the models have significant threshold effects when *l*.*NPLs* are used as threshold variable, and CRBs have a higher threshold value than JSBs.

**Table 3:** Threshold effect tests with threshold variables *l*.*NPLs*_*it*_^,^ *PCR*_*it*_, and *CAR*_*it*_.

	Sample	*l*.*NPLs*_*it*_		*PCR*_*it*_		*CAR*_*it*_	
		Threshold	P-value	Threshold	P-value	Threshold	P-value
***LGR***_***it***_	JSBs	0.016	0.000	2.312	0.000	0.101	0.060
	CRBs	0.031	0.007	3.022	0.010	0.091	0.000
	all	0.010	0.000	2.363	0.000	0.091	0.000
***l*.*LGR***_***it***_	JSBs	0.016	0.000	2.312	0.000	0.090	0.163
	CRBs	0.031	0.003	2.352	0.000	0.091	0.000
	all	0.016	0.000	2.363	0.000	0.091	0.000

*Note*: *P*-values are constructed using 300 bootstraps. We test the threshold effect for JSBs, CRBs, and all the banks in the sample separately.

The results of the threshold regressions are in Table 4, showing that for models of JSBs and all banks in our sample, *β*_0_ is negative and *β*_1_ is positive. This result is consistent with our hypothesis that when *l*.*NPLs* are above the threshold value, banks are more likely to increase risky investment by increasing subprime loans, which could lead to high NPLs. Table 4 also shows that the *NPLs* of all the banks in our sample negatively respond to *ROA* and the *NPSs* of JSBs negatively respond to *ROE*. The results for CRBs show that *β*_0_ is not significant and *β*_1_ is positive, which is also consistent with our hypothesis that when NPLs reach the threshold value, banks tend to increase loans and have high NPLs. The *NPLs* of CRBs respond negatively to *ROA* and *DAR*, but positively to *ROE*. In terms of the response to GDP, the results show that the NPLs of CRBs and all banks in our sample respond negatively to the macroeconomic variable *GDP*_*t*_, which is consistent with our hypothesis that low economic growth could lead to high NPLs.

**Table 4:** Regression with *l*.*NPLs*_*it*_ as the threshold variable.

	*LGR*_*it*_			*l*.*LGR*_*it*_		
	JSBs	CRBs	All	JSBs	CRBs	All
***ROA***_***it***_	0.410 (0.301)	-2.341*** (0.467)	-0.870*** (0.300)	0.252 (0.318)	-2.291*** (0.457)	-0.720** (0.298)
***ROE***_***it***_	-0.087*** (0.019)	0.094*** (0.034)	-0.018 (0.021)	0.081*** (0.020)	0.091*** (0.032)	-0.023 (0.020)
***DAR***_***it***_	0.087 (0.054)	-0.247*** (0.068)	-0.016 (0.046)	0.092 (0.058)	-0.243*** (0.066)	0.003 (0.046)
***GDP***_***t***_	-0.022 (0.018)	-0.038** (0.018)	-0.029** (0.014)	-0.012 (0.020)	-0.038** (0.018)	-0.028** (0.014)
$\beta_{0}\;{LGR}_{it}*I({l.NPLs}_{it}\leq \gamma )$	-0.011*** (0.003)	0.003 (0.004)	-0.014*** (0.002)			
$\beta_{1}\;{LGR}_{it}*I({l.NPLs}_{it}>\gamma )$	0.012*** (0.003)	0.073*** (0.010)	0.007*** (0.003)			
$\beta_{0}\;{l.LGR}_{it}*I({l.NPLs}_{it}\leq \gamma )$				0.012*** (0.003)	-0.0001 (0.003)	-0.014*** (0.003)
$\beta_{1}l.{LGR}_{it}*I({l.NPLs}_{it}>\gamma )$				0.010** (0.004)	0.074*** (0.010)	0.006** (0.002)
***ρ***	0.261	0.328	0.294	0.265	0.328	0.304
***R*^2**	0.648	0.524	0.484	0.613	0.539	0.494
**Observations**	168	228	396	168	228	396

*Note*: standard errors are reported in parentheses. ***, **, and * significant at 1%, 5%, and 10%, respectively.

### Regulatory effect test with *PCR* and *CAR* as threshold variables

To test whether the government requirement for high capital reserves could help mitigate moral hazard behavior, we use *PCR* and *CAR* as threshold variables to examine the effect of government regulations on banks. According to Eq (3), when *PCR*_*it*_ is above the threshold value, the effect of *LGR*_*it*_ on *NPL*_*it*_ is determined by *β*_1_. Banks with *PCR* above the threshold value should have low NPLs, which means that *β*_1_ should be negative and *β*_0_ should be positive. The results of the threshold tests in Table 3 show that all the threshold test results are significant when *PCR* is used as the threshold variable, and CRBs have a higher threshold than JSBs. Table 5 reports the threshold regression results with *PCR* as the threshold variable.

**Table 5:** Regression with *PCR*_*it*_ as the threshold variable.

	*LGR*_*it*_			*l*.*LGR*_*it*_		
	JSBs	CRBs	All	JSBs	CRBs	All
***ROA***_***it***_	0.005 (0.289)	-1.759*** (0.486)	-0.766*** (0.293)	-0.325 (0.282)	-1.624*** (0.460)	-0.785*** (0.287)
***ROE***_***it***_	0.053*** (0.018)	0.050 (0.034)	-0.011 (0.020)	-0.039** (0.018)	0.045 (0.032)	-0.017 (0.020)
***DAR***_***it***_	0.007 (0.052)	-0.094 (0.069)	-0.018 (0.045)	-0.022 (0.052)	-0.060 (0.066)	0.009 (0.044)
***GDP***_***t***_	-0.006 (0.017)	-0.056*** (0.019)	-0.048*** (0.013)	0.028 (0.018)	-0.055*** (0.018)	-0.038*** (0.013)
$\beta_{0}\;{LGR}_{it}*I({PCR}_{it}\leq \gamma )$	0.007** (0.011)	0.012*** (0.004)	0.014*** (0.002)			
$\beta_{1}\;{LGR}_{it}*I({PCR}_{it}>\gamma )$	0.020*** (0.034)	-0.005 (0.004)	-0.008*** (0.003)			
$\beta_{0}\;{l.LGR}_{it}*I({PCR}_{it}\leq \gamma )$				-0.004 (0.003)	0.014*** (0.004)	0.010*** (0.002)
$\beta_{1}l.{LGR}_{it}*I({PCR}_{it}>\gamma )$				-0.030*** (0.004)	-0.009*** (0.003)	-0.013*** (0.002)
***ρ***	0.422	0.384	0.372	0.439	0.428	0.387
***R*^2**	0.669	0.476	0.508	0.686	0.532	0.530
Observations	168	228	396	168	228	396

*Note*: standard errors are reported in parentheses. ***, **, and * significant at 1%, 5%, and 10%, respectively.

The regression results in Table 5 show that *β*_0_ is positive and *β*_1_ is negative, which is consistent with our hypothesis that banks with *PCR* above the threshold value have fewer *NPLs* when they increase the loan growth rate. The significant response of NPLs to the macroeconomic variable *GDP*_*t*_ confirms the hypothesis that low economic growth could result in more NPLs.

The results show that the *NPLs* of CRBs and all banks in our sample respond negatively to *ROA*, and the *NPLs* of JSBs respond negatively to *ROE*. The interpretation of the difference in the response of *NPLs* to *ROA* and *ROE* is that *ROA* takes debt into account, while *ROE* does not.

### Regression with *CAR* as the threshold variable

We use the CAR to test whether regulations based on the Basel Accord help to mitigate moral hazard behavior by banks. A higher CAR implies an adequate capital reserve and less likelihood of having more NPLs, whereas a lower CAR indicates a smaller capital reserve and a higher likelihood of having more NPLs. We first test whether our model, which is shown in Table 3, has a threshold effect. According to Table 3, all the threshold tests in the model, except JSBs with *l*.*LGR*, are significant. The results also show that the threshold values for all kinds of banks are from 0.09 to 0.101. The results of the threshold regressions with *CAR* as the threshold variable are reported in Table 6.

**Table 6:** Regression with *CAR* as the threshold variable.

	*LGR*_*it*_			*l*.*LGR*_*it*_		
	JSBs	CRBs	All	JSBs	CRBs	All
***ROA***_***it***_	0.325 (0.356)	-1.014** (0.433)	-0.183 (0.308)	0.169 (0.354)	-1.228*** (0.457)	-0.303 (0.304)
***ROE***_***it***_	0.104*** (0.022)	0.014 (0.030)	-0.060*** (0.021)	-0.098*** (0.023)	0.022 (0.031)	-0.0523** (0.020)
***DAR***_***it***_	0.028 (0.063)	-0.078 (0.062)	0.007*** (0.045)	0.027 (0.063)	-0.093 (0.064)	0.002 (0.045)
***GDP***_***t***_	-0.006 (0.021)	-0.061*** (0.017)	-0.043*** (0.013)	0.015 (0.022)	-0.060*** (0.018)	-0.038*** (0.014)
$\beta_{0}\;{LGR}_{it}*I({CAR}_{it}\leq \gamma )$	0.026*** (0.011)	0.136*** (0.014)	0.079*** (0.012)			
$\beta_{1}\;{LGR}_{it}*I({CAR}_{it}>\gamma )$	0.0004 (0.004)	0.003 (0.004)	0.002 (0.003)			
$\beta_{0}\;{l.LGR}_{it}*I({CAR}_{it}\leq \gamma )$				0.025*** (0.010)	0.074*** (0.010)	0.055*** (0.007)
$\beta_{1}l.{LGR}_{it}*I({CAR}_{it}>\gamma )$				-0.009*** (0.003)	-0.003 (0.003)	-0.004** (0.002)
***ρ***	0.271	0.405	0.347	0.285	0.380	0.339
***R*^2**	0.516	0.601	0.497	0.530	0.551	0.499
Observations	168	228	396	168	228	396

*Note*: standard errors are reported in parentheses. ***, **, and * significant at 1%, 5%, and 10%, respectively.

Table 6 reports that *β*_0_ is significantly positive. For JSBs, *β*_1_ is positive but not significant. For all the banks in our sample, *β*_1_ is negative, which is consistent with our hypothesis that banks with a *CAR* above the threshold value might have fewer *NPLs* when banks increase the loan growth rate. The significant response of NPLs to the macroeconomic variable *GDP*_*t*_ confirms the hypothesis that low economic growth could result in more NPLs.

The results also show that the *NPLs* of JSBs respond negatively to *ROE*, and the *NPLs* of CRBs respond negatively to *ROA*. For all the banks in our sample, the results show that the *NPLs* respond negatively to *ROE*, but positively to *DAR*.

In a competitive environment, when bank managers decide to lend, they tend to choose projects with low probability of defaulting. JSBs have sound management structures and are innovative in terms of products and services, which makes them more efficient and competitive in managing risk.

For CRBs, the risks of default in lending are partly reduced by the guarantees of local government. The CRBs, which serve small and medium-sized businesses, play an important role in boosting local economic development. The operation of CRBs is closely related to local economic development and tends to be risk aversion. Although CRBs are designated to provide financial support for local projects, they still have pressure to improve their ability to manage risk given the high NPL ratio.

### Robustness analysis

Banks with high NPLs are the result of poor loan decisions made by bank managers. They make lending decisions based on the potential returns and costs of a loan. The returns are determined by the interest rates. The costs are determined by the cost of banking and the risk of default. Credit managers are expected to estimate the risk of default based on the projection of revenues and the cost to offset the losses. we replace LGR with the cost-to-return ratio (CRR) to run the robustness tests. The threshold effect tests in models with *CRR* are shown in Table 7. Unlike the threshold effects in Table 3, the results in Table 10 show that the threshold effect of JSBs becomes insignificant when *CAR* is used as the threshold variable. Table 8 shows the threshold regression results when *l*.*NPLs* is used as the threshold variable to test whether banks with high previous loan loss tend to increase cost inefficiencies, which leads to more *NPLs*. Table 9 shows the threshold regression results when PCR is used as the threshold variable, and Table 10 shows the results when CAR is used as the threshold variable.

**Table 7:** Threshold effect tests with the threshold variables *PCR*_*it*_ and *CAR*_*it*_.

	Sample	*l*.*NPLs*_*it*_		*PCR*_*it*_		*CAR*_*it*_	
		Threshold	P-value	Threshold	P-value	Threshold	P-value
***CRR***_***it***_	**JSBs**	0.019	0.000	2.312	0.000	0.090	0.160
	**CRBs**	0.003	0.100	1.509	0.100	0.091	0.000
	**all**	0.016	0.020	2.259	0.003	0.090	0.050
***l*.*CRR***_***it***_	**JSBs**	0.024	0.000	2.312	0.000	0.101	0.177
	**CRBs**	0.031	0.100	1.509	0.097	0.091	0.000
	**all**	0.017	0.037	2.259	0.000	0.090	0.023

*Note*: *P*-values are constructed using 300 bootstraps. We test the threshold effect for JSBs, CRBs, and all the banks in our sample separately.

**Table 8:** Regression with *l*.*NPLs*_*it*_ as the threshold variable.

	*CRR*_*it*_			*l*.*CRR*_*it*_		
	JSBs	CRBs	All	JSBs	CRBs	All
***ROA***_***it***_	0.099 (0.298)	-1.992*** (0.498)	-0.687*** (0.293)	-0.115 (0.276)	-2.089*** (0.483)	-0.646** (0.305)
***ROE***_***it***_	-0.081*** (0.019)	0.052 (0.035)	-0.024 (0.020)	0.064*** (0.018)	0.070** (0.034)	-0.026 (0.021)
***DAR***_***it***_	0.064 (0.052)	-0.117 (0.071)	-0.013 (0.047)	0.021 (0.049)	-0.179** (0.070)	-0.008 (0.047)
***GDP***_***t***_	0.011 (0.019)	-0.056*** (0.019)	-0.046*** (0.014)	0.040 (0.018)	-0.051*** (0.019)	-0.047*** (0.014)
$\beta_{0}\;{CRR}_{it}*I({l.NPLs}_{it}\leq \gamma )$	-0.043** (0.008)	-0.027*** (0.007)	-0.004*** (0.001)			
$\beta_{1}\;{CRR}_{it}*I({l.NPLs}_{it}>\gamma )$	-0.023*** (0.008)	0.030 (0.002)	0.009*** (0.003)			
$\beta_{0}\;{l.CRR}_{it}*I({l.NPLs}_{it}\leq \gamma )$				0.057*** (0.007)	-0.003* (0.001)	-0.003*** (0.001)
$\beta_{1}l.{CRR}_{it}*I({l.NPLs}_{it}>\gamma )$				0.035*** (0.008)	0.035** (0.004)	0.010** (0.002)
***ρ***	0.391	0.352	0.370	0.511	0.384	0.338
***R*^2**	0.669	0.447	0.468	0.706	0.482	0.471
**Observations**	168	228	396	168	228	396

*Note*: standard errors are reported in parentheses. ***, **, and * significant at 1%, 5%, and 10%, respectively.

**Table 9:** Regression with *PCR*_*it*_ as the threshold variable.

	*CRR*_*it*_			*l*.*CRR*_*it*_		
	JSBs	CRBs	All	JSBs	CRBs	All
***ROA***_***it***_	-0.140 (0.275)	-1.918*** (0.479)	-0.899*** (0.304)	-0.194 (0.256)	-1.931*** (0.481)	-0.889** (0.302)
***ROE***_***it***_	-0.038** (0.018)	0.063* (0.033)	-0.009 (0.021)	-0.033** (0.017)	0.064* (0.481)	-0.010 (0.021)
***DAR***_***it***_	0.029 (0.049)	-0.169** (0.440)	-0.022 (0.047)	0.007 (0.046)	-0.172** (0.070)	-0.017 (0.047)
***GDP***_***t***_	0.015 (0.018)	-0.050** (0.019)	-0.047*** (0.014)	0.036** (0.017)	-0.050* (0.019)	-0.048*** (0.014)
$\beta_{0}\;{CRR}_{it}*I({PCR}_{it}\leq \gamma )$	-0.024*** (0.008)	0.030*** (0.007)	0.008*** (0.002)			
$\beta_{1}\;{CRR}_{it}*I({PCR}_{it}>\gamma )$	-0.040*** (0.008)	-0.003* (0.002)	-0.001*** (0.001)			
$\beta_{0}\;{l.CRR}_{it}*I({PCR}_{it}\leq \gamma )$				0.036*** (0.007)	0.030*** (0.007)	0.009*** (0.002)
$\beta_{1}l.{CRR}_{it}*I({PCR}_{it}>\gamma )$				0.050*** (0.007)	-0.003* (0.001)	-0.001 (0.037)
***ρ***	0.558	0.393	0.361	0.625	0.306	0.368
***R*^2**	0.711	0.488	0.466	0.743	0.486	0.473
**Observations**	168	228	396	168	228	396

*Note*: standard errors are reported in parentheses. ***, **, and * significant at 1%, 5%, and 10%, respectively.

**Table 10:** Regression with *CAR*_*it*_ as the threshold variable.

	*CRR*_*it*_			*l*.*CRR*_*it*_		
	JSBs	CRBs	All	JSBs	CRBs	All
***ROA***_***it***_	0.014 (0.352)	-1.009** (0.426)	-0.595* (0.323)	-0.285 (0.315)	-0.957** (0.419)	-0.562* (0.321)
***ROE***_***it***_	-0.095*** (0.022)	0.013 (0.029)	-0.044** (0.022)	0.067*** (0.020)	0.010 (0.029)	-0.045** (0.022)
***DAR***_***it***_	0.070 (0.061)	-0.065 (0.060)	0.007 (0.049)	0.012 (0.056)	-0.061 (0.059)	0.006 (0.049)
***GDP***_***t***_	0.029 (0.022)	-0.066*** (0.017)	-0.042*** (0.014)	0.066*** (0.021)	-0.066*** (0.016)	-0.043*** (0.014)
$\beta_{0}\;{CRR}_{it}*I({CAR}_{it}\leq \gamma )$	-0.013 (0.011)	0.251*** (0.025)	0.031*** (0.009)			
$\beta_{1}\;{CRR}_{it}*I({CAR}_{it}>\gamma )$	-0.035*** (0.010)	-0.002 (0.001)	-0.003** (0.001)			
$\beta_{0}\;{l.CRR}_{it}*I({CAR}_{it}\leq \gamma )$				0.048*** (0.009)	0.262*** (0.025)	0.039*** (0.009)
$\beta_{1}l.{CRR}_{it}*I({CAR}_{it}>\gamma )$				0.058*** (0.009)	-0.002 (0.001)	-0.003** (0.001)
***ρ***	0.395	0.446	0.383	0.515	0.441	0.356
***R*^2**	0.550	0.614	0.431	0.613	0.628	0.435
**Observations**	168	228	396	168	228	396

*Note*: standard errors are reported in parentheses. ***, **, and * significant at 1%, 5%, and 10%, respectively.

The results in Table 8 show that *β*_0_ and *β*_1_ are both negative for JSBs, which means that banks with previous loan losses increase their costs and have fewer *NPLs*. The interpretation of the results is that JSBs have large capital and highly skilled managers, which helps them to be more cautious and efficient about investment, given large losses.

The results for CRBs and all the banks in our sample show that *β*_0_ is negative and *β*_1_ is positive, which indicates that banks with high previous losses tend to increase costs and have more *NPLs*. These results indicate that CRBs are less efficient about investment and tend to increase costs inefficiently, given large losses, resulting in high *NPLs*.

The results also show that the NPLs of JSBs respond negatively to *ROE*, and the NPLs of CRBs respond negatively to *ROA*. In addition, the NPLs of CRBs respond negatively to *DAR*, which means that high debt could result in fewer NPLs. The results for all the banks in our sample show that NPLs respond negatively to *ROA*.

The results in Table 9 show that *β*_0_ and *β*_1_ are negative for JSBs, which means that JSBs are more cautious about increasing costs and have fewer *NPLs*. The results also show that *β*_0_ is positive and *β*_1_ is negative for CRBs and all the banks in our sample. This result is consistent with our hypothesis that banks with *PCR* above the threshold value become more cautious and are efficient about increasing costs, which results in fewer *NPLs*. Moreover, the results show that NPLs of all banks respond negatively to GDP. Table 9 also shows that the NPLs of JSBs respond negatively to ROE, and the NPLs of CRBs and all the banks respond negatively to ROA. The NPLs of CRBs respond positively to ROE and negatively to DAR.

Because JSBs do not have a significant threshold effect when CAR is used as the threshold variable, we focus only on the models of CRBs and all the banks in our sample. Table 10 shows that *β*_0_ is positive and *β*_1_ is negative for CRBs and all the banks in our sample. The interpretation is that when banks with a high *CAR* increase costs, they become more cautious and efficient about loans, which leads to fewer *NPLs*. In addition, the results show that the NPLs of all banks respond negatively to GDP. The results also show that the NPLs of CRBs respond negatively to *ROA*, and the NPLs of all the banks in our sample respond negatively to both *ROA* and *ROE*.

## Conclusion and policy implications

We use 33 Chinese banks to study the behavior of Chinese banks with a threshold model to test whether government regulations help mitigate moral hazard problems. We find that all the banks with high previous losses tend to increase loans and have more losses. Government regulations help to limit this phenomenon. Banks with PCR and CAR above the threshold value have significantly fewer NPLs when they increase loans. After we split the sample into JSBs and CRBs, we find that both have a significant tendency toward moral hazard, and regulatory policy helps to increase lending efficiency. The results also show that banks with low returns tend to engage in risk-seeking behavior and have more NPLs.

Government regulations could hedge against the negative effect of risk-taking behavior by banks, which strengthens the importance of bank regulations for capital management and risk control. The results also show that NPLs react negatively to economic growth, which confirms the real economic effect.

The risk-seeking investment and less efficient lending by banks distort capital allocation and increase the probability of bad loans, which contributes to an increase in NPLs. Given the inefficiency of market mechanisms in regulating bank behavior, a capital regulation policy by the government is needed to prevent banks from large losses and encourage good capital management. In the long run, policies that promote upgrading in the structure of the economy and production driven by innovation should help to ensure stable economic growth and reduce the number of NPLs.

Given the available data, this paper only focuses on 33 banks, which limits the application of the results. The data for bank management is not available, which makes it impossible to directly estimate how the behavior of bank managers affects the performance of banks. Since the threshold model of Hansen assumes that the threshold variable is exogenous. the efficacy of the regression models could be limited [38]. We will use the structural threshold regression model which allows for endogeneity in threshold variables in our future research [39]. The poor management hypothesis which is proposed by Berger and DeYoung explains why banks choose investment projects with little credit. The data of bank managers are not available, which prevents us from testing the poor management hypothesis in our paper. We will follow the update of the Chinese dataset and collect as much data as we can to test the poor management hypothesis in the future.
